# *Sida hermaphrodita* seeds as the source of anti *- Candida albicans* activity

**DOI:** 10.1038/s41598-019-48712-1

**Published:** 2019-08-22

**Authors:** Kinga Lewtak, Marta J. Fiołka, Paulina Czaplewska, Katarzyna Macur, Zbigniew Kaczyński, Tomasz Buchwald, Ewa Szczuka, Jolanta Rzymowska

**Affiliations:** 1 Department of Plant Anatomy and Cytology, Institute of Biology and Biochemistry, Maria Curie-Skłodowska University, Lublin, Poland; 2 Department of Immunobiology, Institute of Biology and Biochemistry, Maria Curie- Skłodowska University, Lublin, Poland; 3 Laboratory of Mass Spectrometry, Intercollegiate Faculty of Biotechnology, University of Gdańsk and Medical University of Gdańsk, Gdańsk, Poland; 4 Department of Biomedical Chemistry, Laboratory of Structural Biochemistry, Faculty of Chemistry, University of Gdańsk, Gdańsk, Poland; 5 Institute of Material Research and Quantum Engineering, Faculty of Technical Physics, Poznań University of Technology, Poznań, Poland; 6 Chair and Department of Biology and Genetics, Medical University of Lublin, Lublin, Poland

**Keywords:** Antifungal agents, Cellular microbiology

## Abstract

*Sida hermaphrodita* is a perennial herbaceous plant with potential economic importance; however, there is no information about its antimicrobial properties. The aim of our study was to analyze the morphology and metabolic activity of *Candida albicans* cells after exposure to the extract from *S*. *hermaphrodita* seeds, determine its cytotoxicity against human skin fibroblasts and carry out chemical analysis of the extract. Microscopic analysis showed that the crude seed extract (CSE) caused a significant decrease in the metabolic activity of fungal cells, clear cell deformation, and budding disturbances. The analysis of cytotoxicity showed no influence of the extract on the fibroblasts. The CSE and seed extract after dialysis (DSE) were analyzed using electrophoretic, chromatographic, and spectroscopic methods. SDS-PAGE electrophoresis showed the presence of proteins and carbohydrate compounds in the extract. The Raman spectroscopy analysis of the DSE confirmed the presence of proteins, while FTIR analyses revealed the occurrence of albumin-type proteins. The NMR and GC-MS analyses showed the presence of carbohydrates in the seed extract. The MALDI and ESI LC-MS/MS analysis of the CSE and the DSE fractions revealed the occurrence of vicilin-type and plant lipid transfer proteins. The seed extract is a promising formulation to use in *C*. *albicans* infections.

## Introduction

Plants have been a major source of disease curing drugs for thousands of years. The modern pharmaceutical industry has achieved its current level of success as a result of plant-based medicine^1^. More than a half of all modern clinical drugs are of natural origin, for example 60% of anticancer compounds and three quarters of infectious disease drugs are based on natural products^2^.

Virginia mallow (*Sida hermaphrodita*) is a perennial of the *Malvaceae* family. This species is of increased interest due to its economic value e.g. in pollution control^3^, as biomass for energy generation^4^, and as a source of fibers^5–7^. To date, there has not been any data available about the medical or antimicrobial properties of *S*. *hermaphrodita*; however, there are many reports about plants from the *Sida* genus, which contains approximately 200 species of herbaceous plants with described ethnomedicinal usage in treatment of various diseases^8^. One of the best examples is *Sida cordifolia* (L.). This plant is used for healing various diseases because of the content of ephedrine and pseudoephedrine in its leaves and roots^9,10^. Another valuable medicinal herb is *Sida acuta*. This shrub has many applications which have been proven through the isolation of a multitude of compounds, e.g. it is used for fever, headache, skin diseases, diarrhea, and infectious diseases. Other properties of this plant include antiplasmodial, antimicrobial, and antioxidant activities. Alkaloids and steroidal compounds that are supposed to be responsible for antimalarial activity have also been isolated from this species^11,12^.

*Candida albicans* belongs to the physiological human microbiota and at the same time is considered one of the most common human fungal pathogen due to its ability to thrive in many organs^13,14^. The pathogenicity of the *Candida* species is also caused by its adaptability manifested by evading the host immune defense, its ability to adhere, form biofilms, and produce hydrolytic enzymes like proteases, phospholipases, and hemolysin that damage tissues^15^. *C*. *albicans* is the most frequently found species in major fungal infections: superficial types, such as oral candidiasis, and also systemic infections^16^. The increased number of immunocompromised patients, the use of broad-spectrum antibiotics, and transplantations all contribute to the increased incidence of these infections^17^. *C*. *albicans* is a public health concern which has an economic impact due to the costs of care and duration of hospitalization associated with it^18^. Therefore, the search for new and efficient anti*-Candida* drugs has attracted the interests of researchers worldwide.

The aim of our study was to analyze the effects of *S*. *hermaphrodita* seed extract against *C*. *albicans* cells and the chemical characterization of the obtained extract.

## Results

### Analysis of the crude *S. hermaphrodita* seed extract (CSE)

#### Quantification of yeast viability after incubation with the CSE

**Figure 1:**
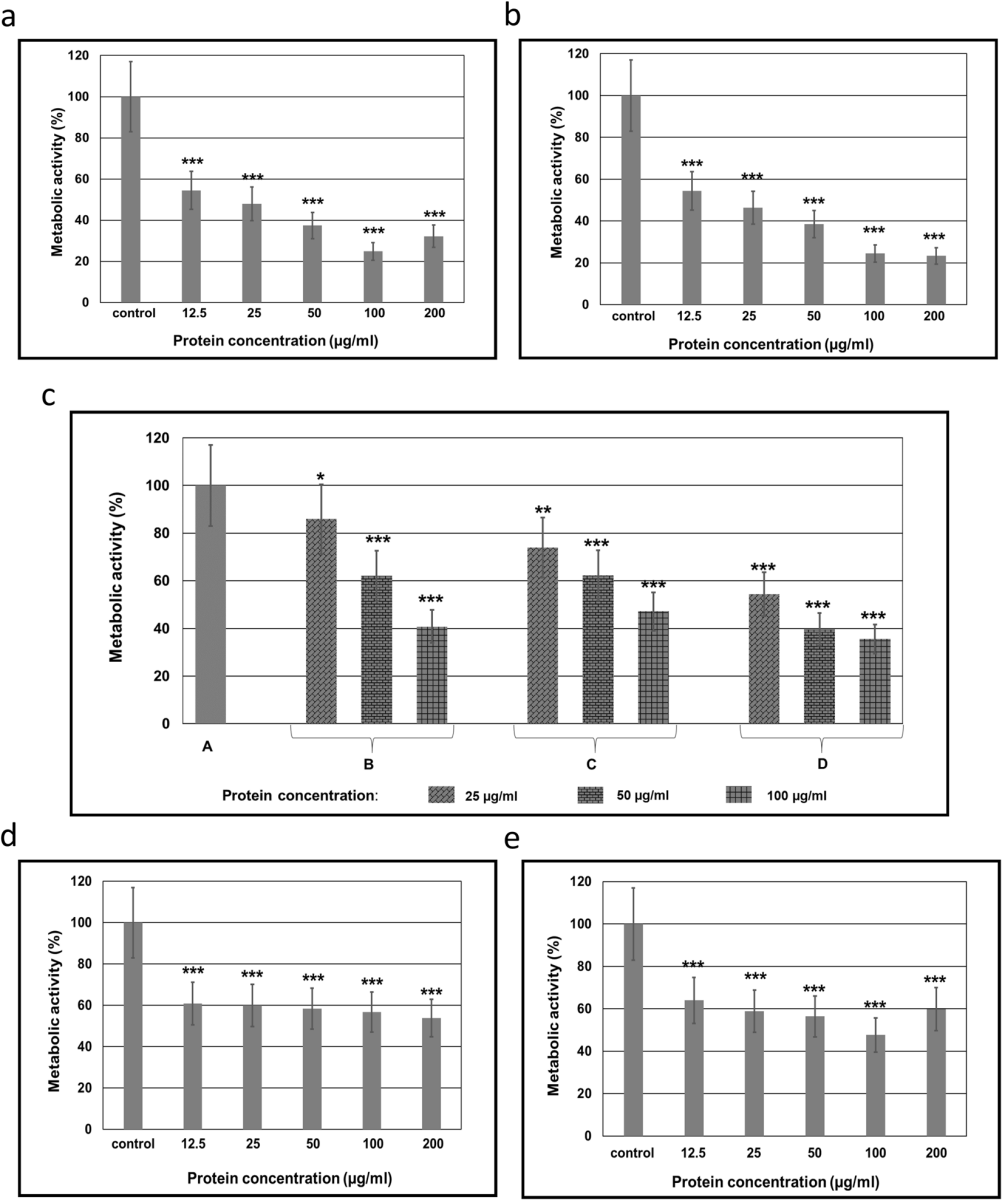
Metabolic activity of *Candida* cells after the incubation (72 h) with the extracts: (**a**) Relative metabolic activity of a wild type *C*. *albicans* cells after the incubation with the crude *S*. *hermaphrodita* seed extract (CSE); (**b**) Relative metabolic activity of a wild type *C*. *albicans* cells after the incubation with the dialysed *S*. *hermaphrodita* seed extract (DSE); (**c**) Relative metabolic activity of wild type *C*. *albicans* cells after the incubation with the dialysed *S*. *hermaphrodita* seed extract fractions. A - *C*. *albicans* control culture; B – *C*. *albicans* cells after treatment with the fraction containing compounds with molecular mass below 30 kDa; C – fraction with compounds with MW in the range of 50–100 kDa; D – fraction with compounds with MW above 100 kDa; (**d**) Relative metabolic activity of *C*. *albicans* ATCC 10231 cells after the incubation with the dialysed *S*. *hermaphrodita* seed extract (DSE); (**e**) Relative metabolic activity of *C*. *krusei* ATCC 6258 cells after the incubation with the dialysed *S*. *hermaphrodita* seed extract (DSE). The results were obtained from 3 independent experiments, ***P < 0.001, **P < 0.01, *P < 0.05 compared to the control group.

The MIC value of the CSE was estimated at 12.5 μg mL^−1^. The incubation of *C*. *albicans* cells with the CSE (for 72 h) at protein concentrations of 12.5, 25, 50, 100, and 200 μg mL^−1^ caused a significant decrease in the relative metabolic activity of fungal cells. After the action of the extract at protein concentration of 12.5 μg mL^−1^ the metabolic activity was diminished by 45.5%, at the concentration of 25 μg mL^−1^ by 52.1%, after 50 μg mL^−1^ – 62.6%, after 100 μg mL^−1^ – 75.2%, and 200 μg mL^−1^ – 67.8%. The differences were statistically significant vs. the control group (Fig. 1a).

#### Morphological changes in C. albicans cells after the action of the CSE observed using DIC

**Figure 2:**
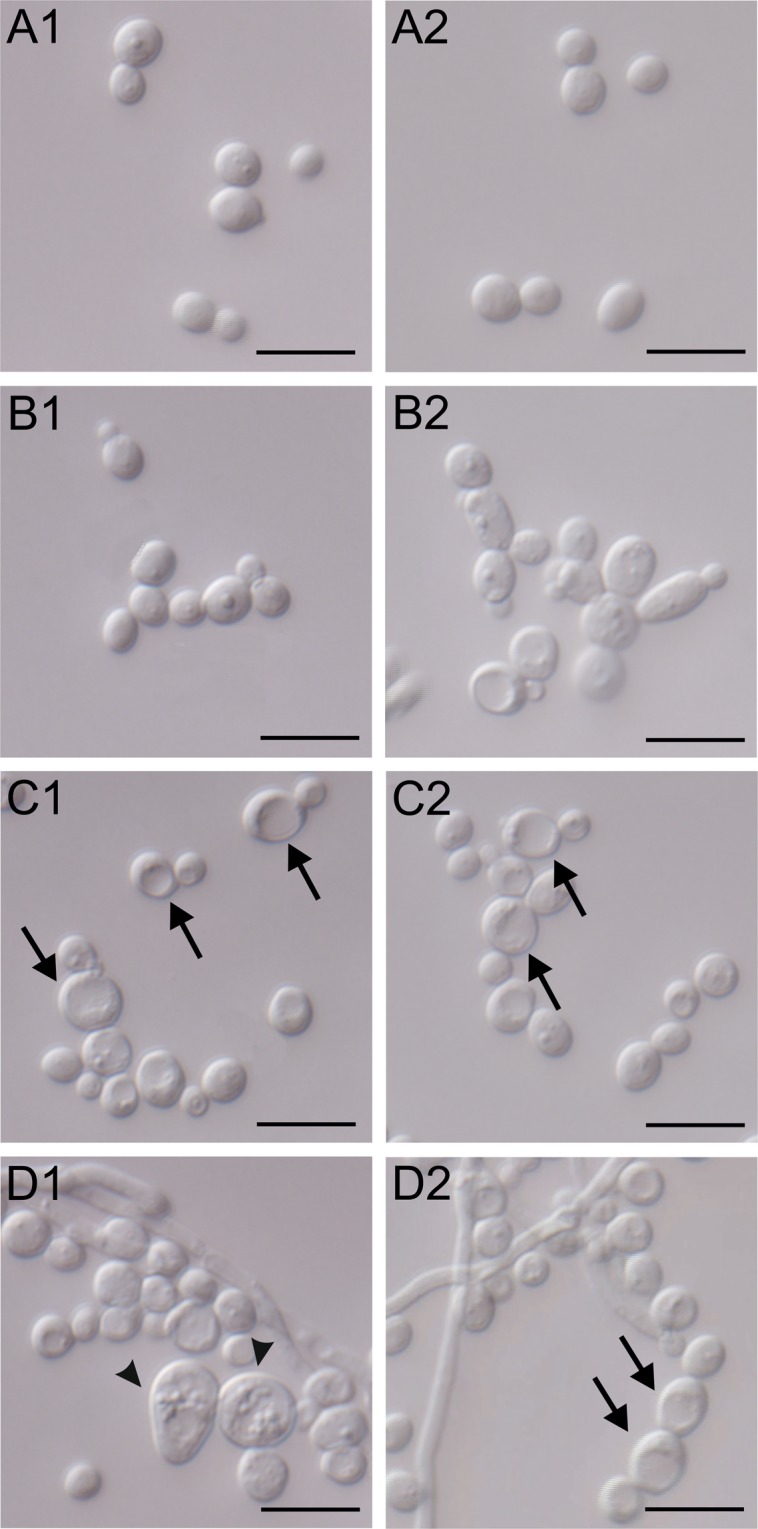
Morphological and cell structure changes in *C*. *albicans* after the action of the crude *S*. *hermaphrodita* seed extract observed using DIC. (**A1–A2**) – *C*. *albicans* control culture; (**B1–B2**) – *C*. *albicans* cells after the treatment with the *S*. *hermaphrodita* seed extract at protein concentration of 25 µg mL^−1^; (**C1–C2**) – at a concentration of 50 µg mL^−1^; (**D1–D2**) - at a concentration of 100 µg mL^−1^. Scale bar represents 10 µm. Arrows indicate cells with enlarged vacuoles, arrowheads indicate cells with granularities on the cell surface.

Observation of yeast cells under the Nomarski-contrast microscope (DIC) allowed comparison of the morphology and the content of the cells. The control cells are oval and have regular shapes with a small cell nucleus visible inside them (Fig. 2A1,A2). The application of the extract at a protein concentration of 25 μg mL^−1^ results in a tendency towards formation of clusters or chains by *C*. *albicans* cells (Fig. 2B1,B2). The use of the 50 μg mL^−1^ extract leads to considerable differences in cell sizes. Enlarged cells exhibit enlarged vacuoles, which occupy at least half of the cell volume indicated by arrows (Fig. 2C1,C2). The greatest differences in yeast cell sizes are visible after the application of the extract at a concentration of 100 μg mL^−1^. In addition to spherical cells - enlarged, oval cells containing vacuoles and granularities on the cell surface were also found (pointed by the arrowheads) in the culture (Fig. 2D1). The medium-sized cells contained various-sized vacuoles indicated by the arrows (Fig. 2D2). Moreover, after treatment with the highest concentration of extract, the presence of filamentous *C*. *albicans* cells was observed (Fig. 2D1,D2).

#### Morphological analysis of C. albicans cells after the action of the CSE using fluorescence microscopy

**Figure 3:**
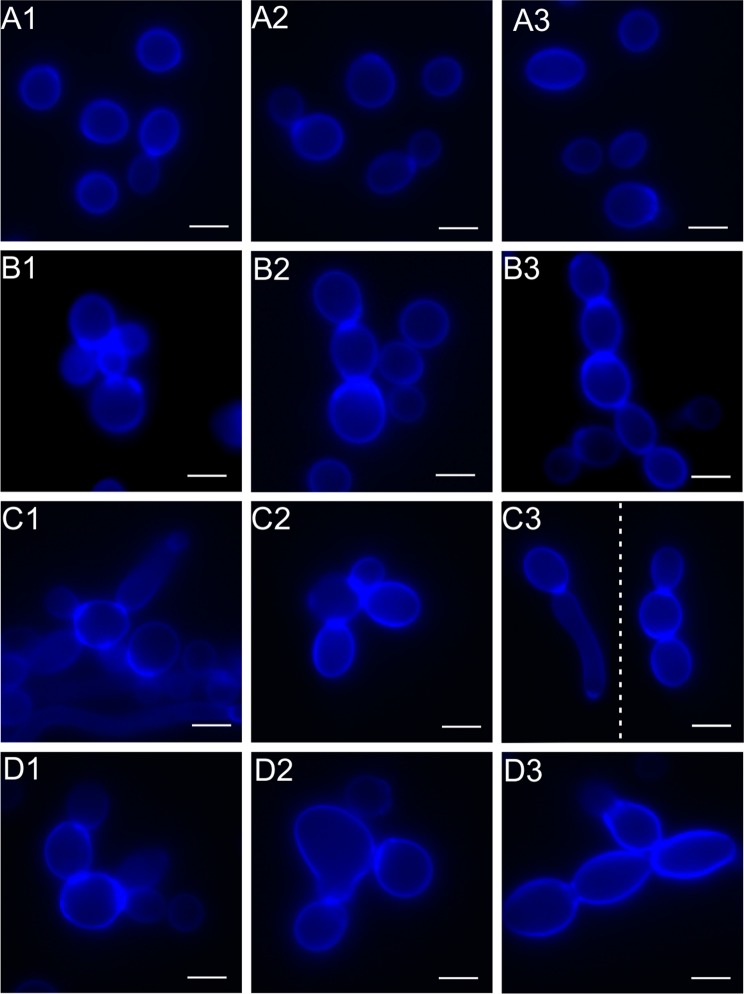
Morphological changes in *C*. *albicans* cells treated with the crude *S*. *hermaphrodita* seed extract observed under a CLSM microscope after staining with Calcofluor White. (**A1–A3**) – *C*. *albicans* control culture; **(B1–B3**) – *C*. *albicans* cells after the treatment with the seed extract at protein concentration of 25 µg mL^−1^; (**C1–C3**) – at a concentration of 50 µg mL^−1^; (**D1–D3**) - at a concentration of 100 µg mL^−1^. Scale bar represents 5 µm. The cell walls of connected cells were irregular in thickness and fluorescence. Cells treated with the extract at the different concentrations showed a tendency to form multicellular chains and abnormal cell configurations.

The use of the Calcofluor White fluorochrome revealed intense blue fluorescence of *C*. *albicans* cell walls. In the control culture, the fluorescence is uniform over the entire cell surface with the exception of the sites of contact with buds. The outlines of the cell walls visible in the fluorescence microscope indicate similar sizes of single cells and their regular, oval shape. Cells of control cultures presented a normal budding pattern (Fig. 3A1–A3). After the treatment with the CSE at a protein concentration of 25 μg mL^−1^, enlargement of the yeast cells is observed (Fig. 3B1–B3). Their cell walls exhibit more intense fluorescence than those in the control cells. The use of the extract at a concentration of 50 μg mL^−1^ in the experiment resulted in the formation of chains composed of several different sized cells (Fig. 3C1-C2). After the treatment with the CSE at a concentration of 100 μg mL^−1^, the cell walls, stained with Calcofluor White, surround substantially enlarged cells. These cells form aggregates composed of a small number of cells (Fig. 3D1-D2).

#### SEM analysis of C. albicans cells after the action of the CSE

**Figure 4:**
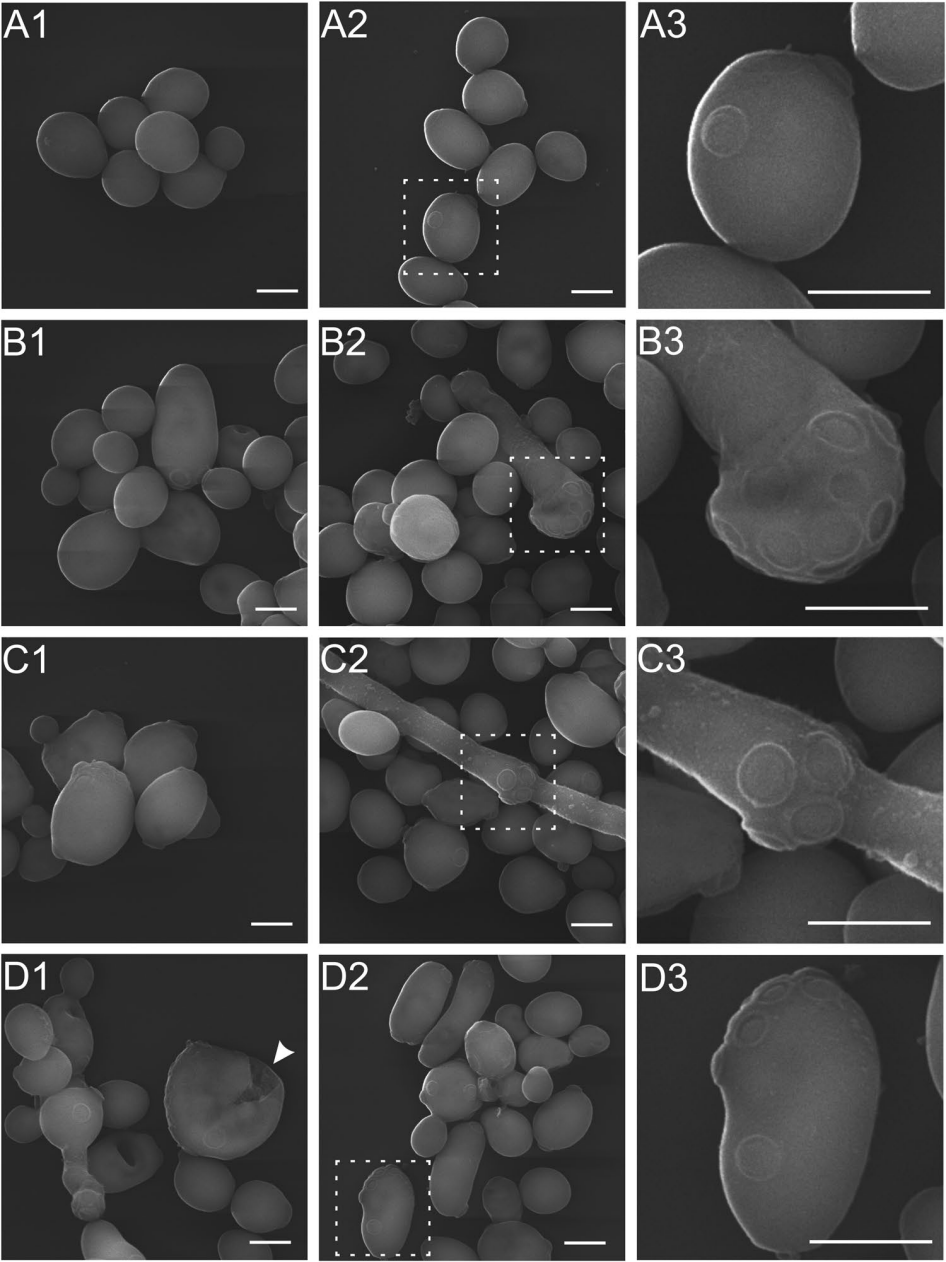
SEM micrographs of: (**A1–A3**) - *C*. *albicans* control culture; (**B1–B3**) – culture after the incubation with the crude *S*. *hermaphrodita* seed extract at protein concentration of 25 µg mL^−1^; C – at a concentration of 50 µg mL^−1^; (**D1–D3**) - at a concentration of 100 µg mL^−1^. (**A3**,**B3**,**C3** and **D3**) shows enlarged sections in the squares from images (**A2**, **B2**, **C2** and **D2**); the arrowhead indicates a cell with a ruptured cell wall. Scale bar represents 2 µm.

*C*. *albicans* cells treated with the CSE exhibit distinct concentration-dependent morphological changes. In the control, single similar sized yeast cells observed under the scanning microscope have regular, oval shapes. The smooth surface of some of the cells bear scars or convexities, suggesting initiation of budding (Fig. 4A1–A3). After the treatment with the CSE at a protein concentration of 25 μg mL^−1^, the cell size is clearly varied. Besides the more numerous spherical, small-sized cells, there are two- or three-fold larger cells characterised by an oval or elongated shape. The surface of both large and small cells is smooth (Fig. 4B1,B2). Abnormal grouping of bud scars located at one pole of the elongated cell was also observed (Fig. 4B2,B3). The extract at a concentration of 50 μg mL^−1^ caused drastic changes in the yeast cell morphology (Fig. 4C1). Moreover, numerous bud scars were seen on one side of the hyphal septum, indicating disorders in yeast budding (Fig. 4C2,C3). In comparison with the yeast cells in the control, cells treated with the CSE at a concentration of 100 μg mL^−1^ exhibit substantial disproportions in their size and shape. Furthermore, the cell walls of both the enlarged and regular-sized cells are irregular, which causes cell deformation. After the treatment with the CSE at a concentration of 100 μg mL^−1^, beside the regularly shaped, oval cells with a size typical of *C*. *albicans*, there is an inflated cell with a relatively large size and with a wall rupture (pointed by arrowhead) (Fig. 4D1). Among the regular-shaped cells, single cells produce hyphae. The enlarged cells are surrounded by a non-uniformly thick cell wall with recesses or discontinuities (Fig. 4D1). The majority of the cells are distinctly enlarged, and some of them are budding or bear scars on their surface, which indicates intensive polar budding (Fig. 4D2,D3).

#### AFM analysis of C. albicans cells after the action of the CSE

**Figure 5:**
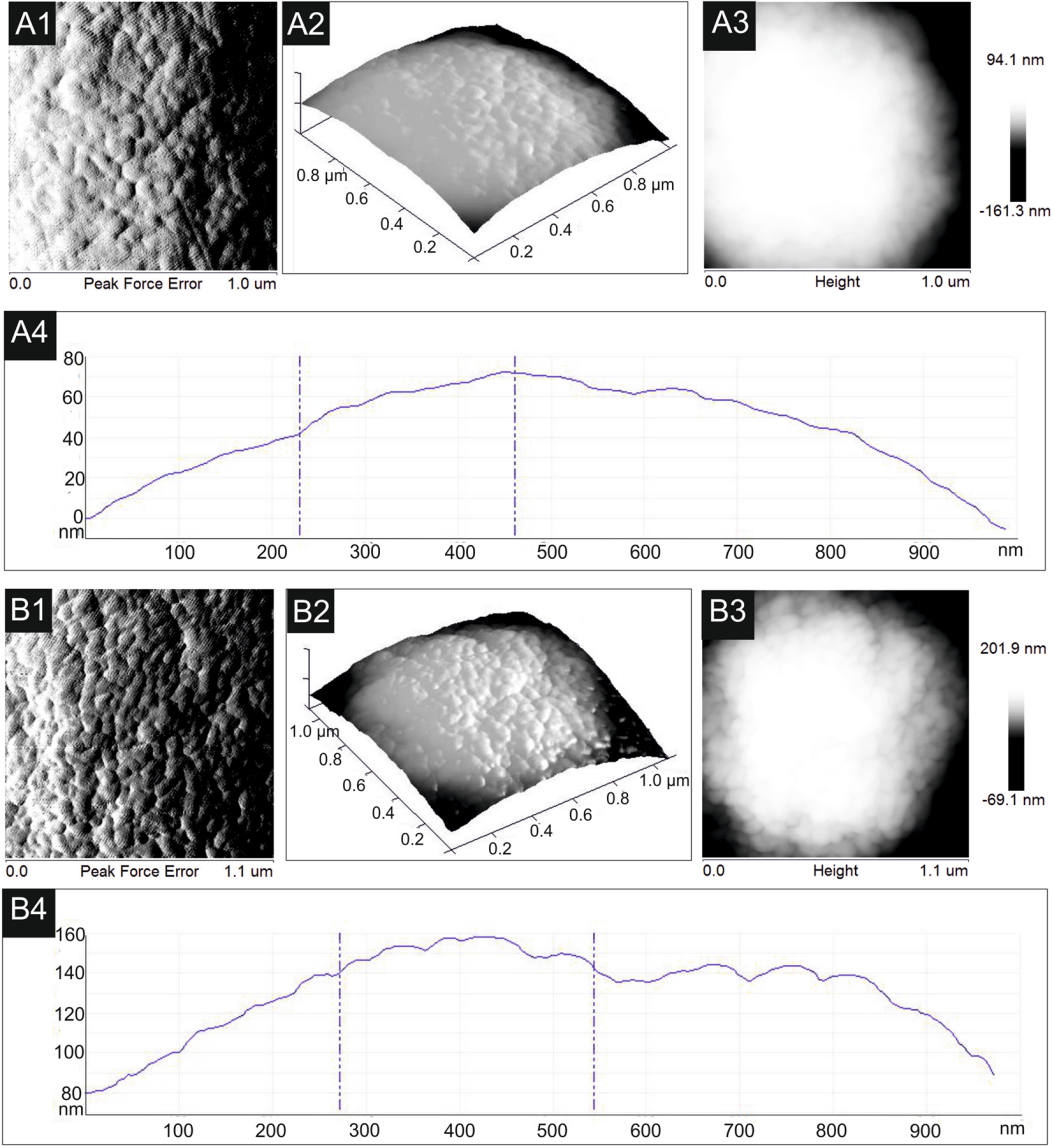
AFM images of (**A1–A4**)– cell surface of *C*. *albicans* cell of control culture; (**B1**–**B4**)– cell surface of cells after the treatment with the crude *S*. *hermaphrodia* seed extract at protein concentration of 100 µg mL^−1^. (**A1,B1**) – peak force error images of surface fragments of yeast cells; (**A2**,**B2**) – three-dimensional representation of (**A1** and **B1**); (**A3**,**B3**),– height images of cell surfaces; (**A4**,**B4**) – height profiles of cell surfaces. After exposure to the extract, the cell surface is more irregular in shape.

AFM analysis of the *C*. *albicans* cells after incubation with the CSE at a protein concentration of 100 ug mL^−1^ showed changes in the cell surface, in comparison to the untreated control cells (Fig. 5A1–A4). The profile of the cell surface after incubation with the extract was less regular than that of the control cells; cavities were clearly visible (Fig. 5B1–B4). Moreover, the surface roughness of the control *C*. *albicans* cells was lower (average roughness was 24.2 nm) than the average roughness (39.7 nm) of the surfaces of yeast cells after the treatment with the CSE at a concentration of 100 ug mL^−1^.

#### MALDI and ESI LC-MS/MS analysis of the CSE

**Figure 6:**
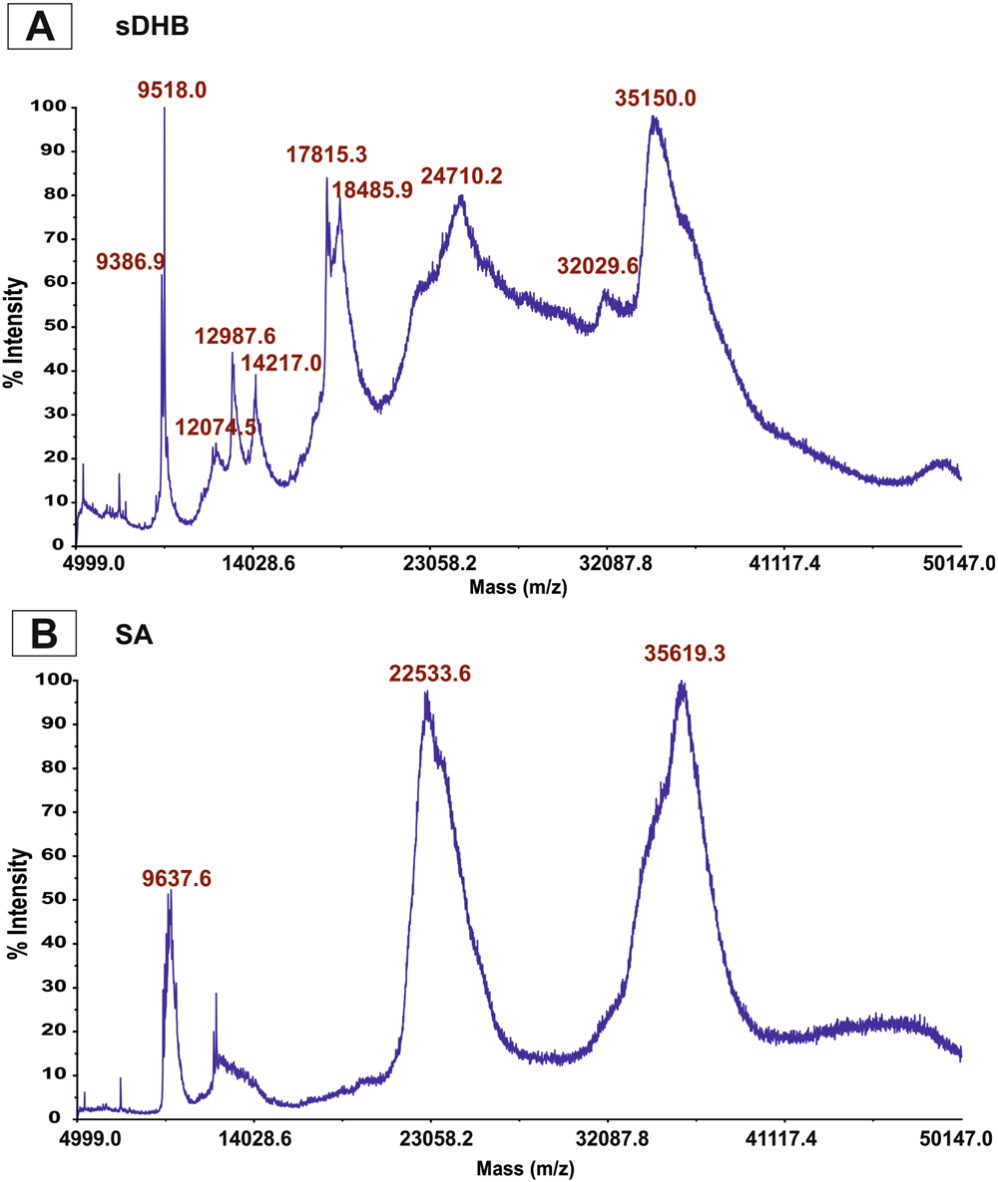
MALDI MS analysis of intact spectra of the crude *S*. *hermaphrodita* seed extract (CSE): (**A**) Linear middle mass mode sDHB matrix; (**B**) Linear middle mass mode SA matrix.

Intact MALDI TOF mass spectra registered in both matrices (Fig. 6) confirmed the proteinaceous character of the sample. Both spectra are a fingerprint for the protein extract from *S*. *hermaphrodita* seeds. The main components are represented by the m/z signals located between 9 and 50 kDa. The sDHB matrix revealed the presence of several dozen proteins (Fig. 6A) and in the case of sinapinic acid, two main broad m/z signals (22.5 kDa and 35.6 kDa) are present in the spectrum (Fig. 6B).

At this level of analysis, we were unable to obtain more information. To better understand the protein composition of the tested extract and its obtained fractions, a classical proteomic analysis was carried out and Trypsin was used to digest proteins after reduction with dithiothreitol (DTT) and alkylation with iodoacetamide (IAA). Tryptic peptides were then analyzed using LC-ESI-MS/MS. The Malvales database (Uniprot) was searched because the protein database for *S*. *hermaphodita* is very poor. The analysis of the seed extract showed the presence of 123 proteins, of which approximately 70 were identified (Table. 1. Suppl.).

#### NMR analysis of the CSE

**Figure 7:**
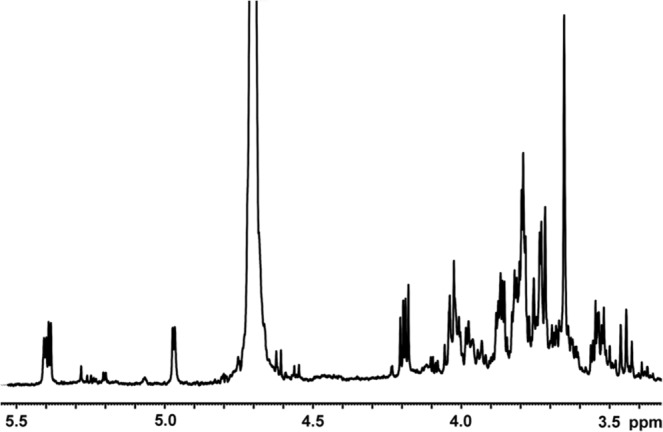
^1^H NMR spectrum of crude *S*. *hermaphrodita* seed extract. Signals in the range of 3–5.5 ppm show the carbohydrate content in the sample.

NMR analysis of the CSE showed the carbohydrate content in the sample as indicated by the signals in the range of 3.4–5.5 ppm (Fig. 7). There are characteristic proton anomeric signals of monosaccharides at 4.98 ppm, 5.39 ppm and δ 5.41 ppm, as well as ring proton signals in the range of 3.4–4.2 ppm.

#### Cytotoxic activity

The MTT assay showed no cytotoxic activity (0%) of the CSE at a protein concentration of 100 µg mL^−1^ after incubation for 72 hours. Observations under the microscope showed no cytotoxic or cytopathic effect on fibroblasts after incubation with the *S*. *hermaphrodita* extract at a concentration of 100 µg mL^−1^ for 72 hours. The cells treated with the extract exhibited normal morphology, similar to the control cells.

### Analysis of the S. hermaphrodita seed extract after dialysis (DSE)

#### Quantification of yeast viability after incubation with the DSE

The MIC for all analyzed strains: the clinical *C*. *albicans* isolate, *C*. *albicans* ATCC 10231, and *C*. *krusei* ATCC 6258 was 12.5 μg mL^−1^. Incubation of cells of a wild type *C*. *albicans*, *C*. *albicans* ATCC 10231, and *C*. *krusei* ATCC 6258 with the DSE containing compounds with molecular mass above 14 kDa (for 72 h) at protein concentrations of 12.5, 25, 50, 100, and 200 μg mL^−1^ also caused a significant decrease in the relative metabolic activity of fungal cells.

After the action of the extract at a protein concentration of 12.5 μg mL^−1^ the metabolic activity of a wild type *C*. *albicans* cells was diminished by 45.6%, at a concentration of 25 μg mL^−1^ by 53.7%, after 50 μg mL^−1^ – 61.5%, after 100 μg mL^−1^ – 75.5%, and 200 μg mL^−1^ – 76.7% (Fig. 1b). The DSE at all protein concentrations reduced the metabolic activity of *C*. *albicans* ATCC 10231 cells at a similar level. The DSE at a protein concentration of 12.5 μg mL^−1^ reduce the metabolic activity by 39.2%, at the concentration of 25 μg mL^−1^ by 40.03%, after 50 μg mL^−1^ – 41.6%, after 100 μg mL^−1^ – 43.2%, and 200 μg mL^−1^ – 46,2%. The differences were statistically significant vs. the control group (Fig. 1d). The relative metabolic activity of *C*. *krusei* ATCC 6258 cells after incubation with the DSE at a protein concentration of 12.5 μg mL^−1^ was diminished by 36%, at the concentration of 25 μg mL^−1^ by 41%, after 50 μg mL^−1^ – 43.6%, after 100 μg mL^−1^ − 52.3% and 200 μg mL^−1^ −40% (Fig. 1e).

#### Microscopic analysis of C. albicans cell death after the DSE treatment

**Figure 8:**
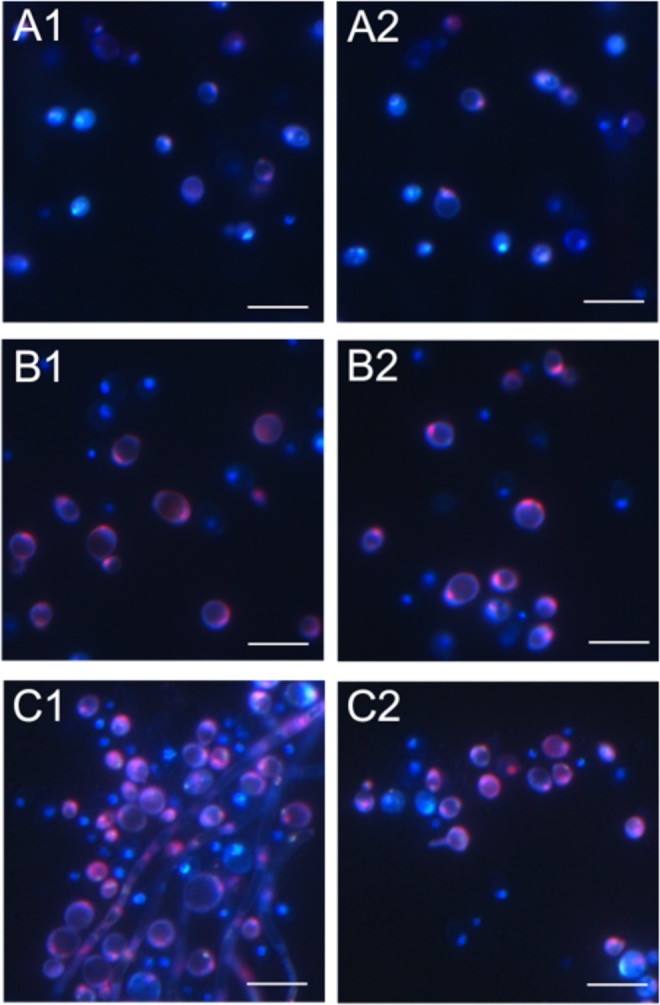
CLSM imaging of necrosis of *C*. *albicans* cells after the incubation with the DSE. (**A1–A2**) - *C*. *albicans* control culture; (**B1–B2**) – *C*. *albicans* cells after the treatment with the DSE at protein concentration of 50 µg mL^−1^; (**C1**–**C2**) – at a concentration of 100 µg mL^−1^. Scale bar represents 10 µm. Necrotic cells are stained pink.

Untreated control *C*. *albicans* cells showed clearly blue fluorescence with strongly marked nuclei (Fig. 8A1,A2). After incubation of *C*. *albicans* cells with the DSE at a concentration of 50 and 100 μg mL^−1^ necrotic cells were observed (Fig. 8B1-C2).

#### Electrophoretic analysis

**Figure 9:**
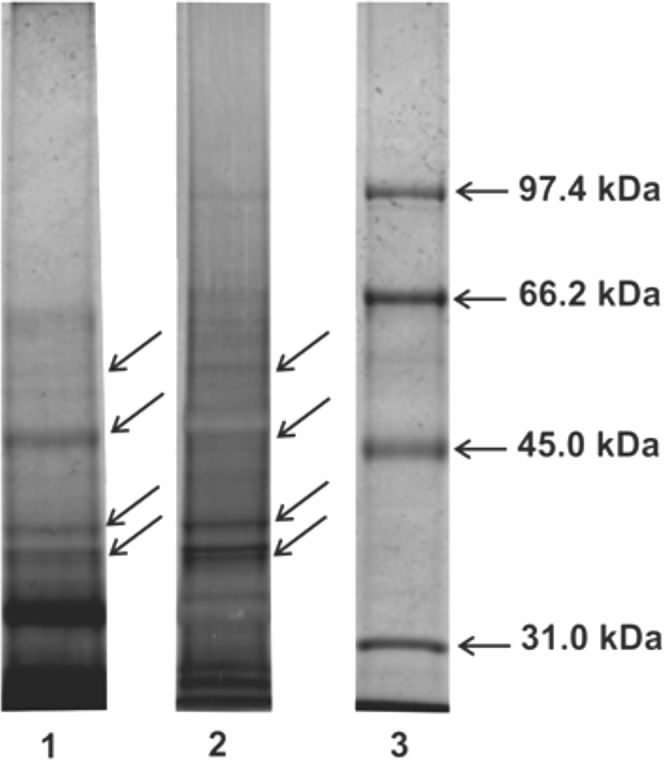
Electrophoretic analysis of DSE using SDS/ PAGE electrophoresis. 1 – compounds stained with silver nitrate, 2 – protein bands stained with Coomassie Brilliant Blue R-250 (Sigma), 3 – molecular weight markers (Bio-Rad). Analyses were performed in 10% polyacrylamide gels. Stained bands were indicated by arrows.

The gels after SDS/PAGE electrophoresis of the DSE were stained with Coomassie Brilliant Blue R-250 and silver nitrate for detection of protein and carbohydrate compounds respectively. Staining showed multiple bands ranging from 38 to 55 kDa for protein and carbohydrate. The most intense bands for both proteins and carbohydrates compounds with molecular mass around 55 kDa, 46 kDa, 40 kDa, 38 kDa were visible on the gel. The obtained results prove the presence of protein-sugar compounds in the analyzed extract (Fig. 9).

#### Raman spectroscopy analysis of the proteins from the DSE

**Figure 10:**
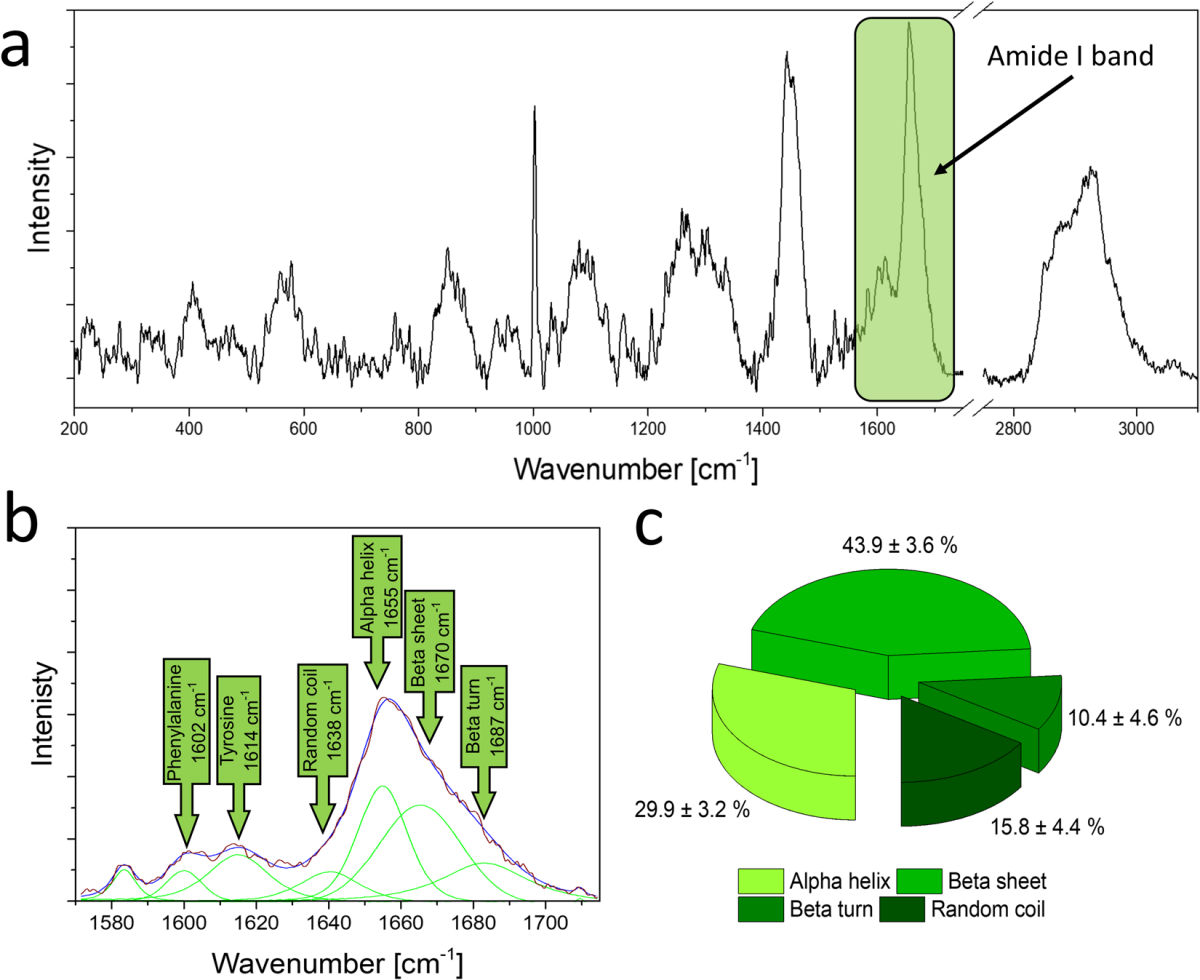
Raman spectroscopy analysis of the DSE. (**a**) Raman spectrum of the DSE with the selected amide I band; (**b**) example of the deconvolution of the amide I band; (**c**) percentage content of the proteins in the study material with a given secondary structure.

Raman spectroscopy is an effective tool for the determination of the protein composition and structure and distinguishing the type of the secondary structure such as the alpha helix, beta sheet, or beta turn^19–23^. Therefore, this spectroscopic method was used to indicate the protein secondary structure of the DSE. Figure 10 presents an example of the Raman spectrum of proteins contained in the study material (A). The amide I band labelled in the Raman spectrum was used to determine the secondary protein structure. To indicate the percentage amount of a particular type of secondary protein structure, the curve-fitting process of the amide I band was applied. This process allowed the determination of the intensity of overlapping bands. The curve-fitting process in the amide I band region is presented in Fig. 10b. The bands related to the alpha helix and beta-sheet structure are located at 1655 cm^−1^ and 1670 cm^−1^, respectively. The band assigned to the unordered structure (random coil) is detected at 1638 cm^−1^, whereas the band representing the beta turn structure is visible at 1687 cm^−1^. Furthermore, the bands at 1602 cm^−1^ and 1614 cm^−1^ are associated with phenylalanine and tyrosine ring modes, respectively. The intensity of the bands in the amide I region allowed accurate estimation of the alpha helix, beta sheet, beta turn, and random coil content in the analyzed material. Figure 10c presents the percentage amount of protein in a particular secondary structure in the study material determined by the analysis of the band intensity in the analyzed spectral region.

#### Fourier Transform Infrared Spectroscopy analysis of the DSE

**Figure 11:**
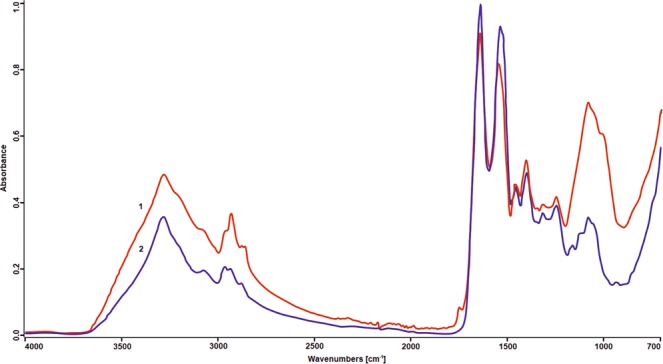
ATR-FTIR analysis of the DSE. 1 - spectrum of the DSE; 2 – spectrum of the albumin from chicken egg white. Spectrum of the DSE showed 95% similarity to the spectrum of the albumin according to ATR Pharmaceutical ST Japan.

The FTIR analysis showed that the DSE spectrum exhibited high similarity to the spectrum of egg albumin protein with a hit quality index (HQI) of 950 according to the ATR-FTIR Pharmaceuticals Library (©S.T. Japan, ©Nicodom) (Fig. 11).

#### GC-MS analysis of the DSE

Chemical analysis of the seed extract in terms of carbohydrate content revealed the presence of glucose and galactose in a ~1:1 ratio.

### Analysis of fractions obtained from the dialysed S. hermaphrodita seed extract (DSE fractions)

#### Quantification of yeast viability after incubation with the DSE fractions

Incubation of *C*. *albicans* cells with the DSE fractions (for 72 h) at protein concentrations of 25, 50 and 100 μg mL^−1^ caused a significant decrease in the relative metabolic activity of fungal cells. After the action of the DSE fraction containing compounds with molecular weight below 30 kDa (14–30 kDa) the metabolic activity was diminished by 14% at a protein concentration of 25 μg mL^−1^, 37.9% at 50 μg mL^−1^, and 59.2% at 100 μg mL^−1^. In the case of the fraction containing compounds with molecular weight in the range of 50–100 kDa the metabolic activity of fungal cells was diminished by 26.1% at a protein concentration of 25 μg mL^−1^, 37.9% at 50 μg mL^−1^, and 52.9% at 100 μg mL^−1^. In turn, the DSE fraction containing compounds with molecular weight above 100 kDa caused a decrease in the metabolic activity of *C*. *albicans* cells by 45.7% at a protein concentration of 25 μg mL^−1^, 60.5% at 50 μg mL^−1^, and 64.4% at 100 μg mL^−1^. The differences were statistically significant vs. the control group (Fig. 1d).

#### LC-ESI-MS/MS analysis of the DSE fractions

Separation of the DSE into fractions facilitated analysis of the content and identification of proteins present in the extract. The analysis revealed the presence of 40 proteins (30 identified) in the fraction containing compounds with molecular mass below 30 kDa, 200 proteins (164 identified) in the fraction containing compounds with molecular mass in the range of 50–100 kDa, and 214 proteins (175 identified) in the fraction containing compounds with a molecular mass above 100 kDa (data are presented in Table 2. Suppl.).

Among the numerous functional and structural proteins, those identified as lipid transfer proteins and vicilins deserve special attention (Table 1). The lipid transfer protein was present in all three DSE fractions, while vicilins were present in the fractions containing compounds with molecular mass in the range of 50–100 kDa and compounds with molecular mass above 100 kDa. These proteins act in the plant mainly as storage proteins but they can also be involved in plant protection processes.

**Table 1:** List of proteins identified as lipid transfer protein and vicilin present in the fractions of dialysed *S*. *hermaphrodita* seed extract provided with their identification parameters: Unused ProtScores, numbers of peptides (95% confidence), and percent of sequence coverage.

N	Name	Accession #	Species	Peptides (95%)	% Cov
**Fraction containing compounds with molecular mass below 30 kDa**					
1	Plant lipid transfer protein/Par allergen	A0A1R3GHN2	9ROSI	2	64
2	Non-specific lipid-transfer protein	U5HU82	GOSBA	2	32,5
**Fraction containing compounds with molecular mass in the range of 50–100 kDa**					
1	uncharacterized protein LOC107929052 (BLAST Vicilin GC72-A 98.8%)	A0A1U8LMX7	GOSHI	49	38,8
2	vicilin-like seed storage protein At2g28490	A0A1U8P4G8	GOSHI	18	29,2
3	vicilin-like seed storage protein At2g18540	A0A1U8K3C6	GOSHI	3	22,5
4	non-specific lipid-transfer protein-like protein At5g64080	A0A1U8NKZ7	GOSHI	2	22,1
5	Seed storage protein vicilin A (Fragment)	G3M3J4	GOSHI	34	48,2
**Fraction containing compounds with molecular mass above 100 kDa**					
1	uncharacterized protein LOC107929052 (BLAST Vicilin GC72-A 98.8%)	A0A1U8LMX7	GOSHI	84	34,8
2	vicilin-like seed storage protein At2g28490	A0A1U8P4G8	GOSHI	29	35,8
3	vicilin-like seed storage protein At2g18540	A0A1U8K3C6	GOSHI	6	35
4	vicilin GC72-A	A0A1U8LQ34	GOSHI	58	43,4

## Discussion

Among all fungal pathogens, *C*. *albicans* has gained an infamous reputation. Under favourable conditions associated with several risk factors, this normally harmless commensal fungus can become an opportunistic source of onerous, recurrent candidiasis or even life-threatening candidemia such as bloodstream and internal organ infections^24^. Taking these data into consideration, the impact of *C*. *albicans* virulence on the significant increase in mortality among patients should be emphasized. This situation leads to a search for new and effective antimicrobial agents, and plants can be a key source for the discovery of new drugs^25^.

Medicinal plants are an important source of new chemical substances with potential antimicrobial and therapeutic effects. Literature data have shown that the vegetative organs of plants are the most popular source of active compounds; however, seeds are increasingly becoming a subject of research interest. The anti-*Candida* activity of both aqueous and alcoholic seed extracts has recently been described by many researchers^26–30^.

In this paper, we studied the antifungal action of an extract from *S*. *hermaphrodita* seeds against *C*. *albicans*. This kind of *S*. *hermaphrodita* extract has never been evaluated as a potential anti-*Candida* agent before. This is the first report on the anti- *C*. *albicans* activity and chemical analysis of the *S*. *hermaphrodita* seed extract.

Our investigations showed that crude *S*. *hermaphrodita* seed extract (CSE) possesses effective antifungal activity. It reduces *C*. *albicans* metabolic activity by half at a protein concentration of 25 μg mL^−1^, and by 75% at a concentration of 100 μg mL^−1^. The changes observed in *C*. *albicans* cells after incubation with the CSE involved an increase in cell size, enlargement of the vacuoles, formation of multicellular chains, deformation of the shape, and thickening of the cell wall. SEM imaging of *C*. *albicans* cells treated with the seed extract revealed damage to the cell wall after exposure to the extract at the highest protein concentration used (100 µg mL^−1^). Additionally, we observed that the seed extract caused disturbances in budding of *C*. *albicans* cells visible as multiple polar budding of blastospores and hyphae. The three-dimensional and height AFM images showed changes in the structure of the *C*. *albicans* cell envelope and an increase in the surface roughness after the CSE treatment.

Similar morphological effects on *C*. *albicans* cells were observed after incubation of yeast cells with amphotericin B at a concentration of 0.25 µg mL^−1^ and 0.5 µg mL^−1^. Antibiotic caused enlargement of whole cells and vacuoles, cell division disorders visible as chain formation. These results were presented in our previous publication^31^. Enlarged vacuoles and irregular budding on the *C*. *albicans* cell surface were observed in closantel drug - treated *C*. *albicans* cells^32^. *C*. *albicans* cell surface abnormalities and burst cells were confirmed after exposure to equol, i.e. an isoflavandiol present in soybean^33^. After using AFM, Tyagi and Malik^34^ observed similar changes in the *C*. *albicans* cell envelope after application of lemon grass essential oil. Moreover, an increase in cell surface roughness after treatment with amphotericin B, fluconazole, and plant defensin *Psd1* was also noted by Silva^35^.

The hallmark feature of *C*. *albicans* cells is the ability to create different morphological forms. The occurrence in two or even three co-existing forms (yeast, hyphal, and pseudohyphal forms) and attempts to explain this phenomenon have been the subject of numerous, detailed studies^36^. All three morphological forms can occur in vegetative cultures^37^, but this phenomenal plasticity determines the virulence of the yeast^38^. As far back as three decades ago, it was reported that the change in the type of growth of the oval single cell into long hyphae can be a response to nutrient stress^39^. Besides many nutritional constituents influencing this transition, e.g. serum or N-acetylglucosamine (GlcNAc), chemical agents or physical factors participate in the process as well. The wide range of these factors, e.g. neutral pH, high temperature, nutrient starvation, hypoxia, CO_2_, and adherence, is considered by^38^. Plant extracts are other critical factors of transition processes affecting the morphology of *C*. *albicans*^40^.

During our experiments it was observed that *Candida* cells took two morphological forms: blastoconidia and filamentous cells. Single-celled, oval forms are the most common and are typical forms of yeast. In our experiments, this form was present in all control cultures. In turn, the hyphae forms appeared in a *C*. *albicans* culture subjected to treatment with the crude seed extract at a concentration of 50 μg mL^−1^ and higher. It can be assumed that the formation of hyphae results from an increase of the extract concentration in the culture, which is a stress factor. Despite the very important and broad investigations of this specific phenomenon that have been conducted by researchers, the transformation and appearance of such diverse morphological forms in *C*. *albicans* still remain an enigma and undoubtedly provides an incentive and challenge for future research.

The results obtained leads to the conclusion that the *C*. *albicans* cell wall is a potential target for the antifungal activity of the active components of the *S*. *hermaphrodita* seed extract. The intact cell wall that surrounds fungal cells is essential for their survival^41^. Therefore, all kinds of changes in the cell wall that can affect the cell resistance to agents used in antifungal therapies can be valuable for elimination of pathogenic yeasts.

In our research the MALDI TOF/TOF and LC-ESI-MS/MS analysis of the crude seed extract (CSE) showed the presence of many functional, structural and storage proteins characteristic for seeds. Proteins identified as vicilin and lipid-transfer protein belonging to plant antimicrobial peptides were found. The NMR analysis showed the presence of carbohydrates in the CSE, and further GC-MS analysis revealed the presence of glucose and galactose.

The dialysed seed extract (DSE) decreased the metabolic activity of *C*. *albicans* cells at the same level as the CSE, which indicates the presence of active compounds in the fraction obtained after dialysis of the extract. Staining of the bands after electrophoretic separation of the DSE showed the presence of both proteins and carbohydrates. The Raman spectroscopy confirms the occurrence of proteins in the DSE. Moreover, this method indicates the percentage content of a particular secondary protein structure. The FTIR analysis of the DSE showed a high similarity of the spectrum obtained to the albumin spectrum, which indicates the presence of chemical bonds characteristic for these types of proteins.

In our research the highest antifungal activity was observed after the action of the fraction containing compounds with molecular mass above 100 kDa. The LC-ESI-MS/MS analysis of this fraction revealed the presence of the largest number of vicilin-type peptides in comparison to other fractions. Antifungal activity of this group of proteins was analyzed in many reports^42,43^. Wang and collaborators^44,45^ analyzed an extract from *Malva parviflora* (*Malvaceae*) and described proteins homologous to cotton vicilins and seed storage protein 2 S albumin exhibiting activity against *Fusarium graminearum* and *Phytophthora infestans*.

The results of our research correspond with the data reported by other authors. Vicilins are described as oligomers with molecular mass of 150–170 kDa, consisting of different subunits by Vieira Bard *et al*.^43^. Moreover, vicilins can occur in the form of glycosylated trimeric clusters and show a high degree of heterogeneity^46^. There are also known derivatives of vicilins: a vicilin-like glycoprotein from *Nicotiana sylvestris*^46^ and a low molecular weight vicilin-like glycoprotein from *Citrullus lanatus* seeds^47^. He and collaborators^48^ revealed the presence of vicilin GC72-A in the cotton seed extract - a plant belonging to the *Malvaceae* family. The same protein was detected in the high molecular weight fraction analysed in our experiments.

It is well known that plant extracts are used in pharmacology as supplements and medicines available in pharmacies. For example, the Pro-Uro supplement containing a cranberry (*Vaccinium macrocarpon*) fruit extract supports the functioning of the urinary tract. There are known medicines based on several plant extracts, such as Venoforton® used in peripheral circulation disorders, and Iberogast® applied for gastrointestinal discomfort. Sylimarol, containing sylibum husk dry extract from *Silybum marianum* is a widely used drug that protects and regenerates the liver. The most popular of medicinal preparations based on seed extracts, is the ‘Grapeseed extract’ used for blood circulation problems due to its antioxidant properties^49^. Natural plant products can also be used as alternative anti-*Candida* drugs^50^.

Although *Candida* are the leading agent of opportunistic infection in patients with immune disorders, the number of effective antifungal drugs is still limited due to the resistance of *Candida* strains to the used antimycotics. Factors that predispose to candidiasis include immunosuppression, steroids treatment and invasive medical procedures, long-term antibiotic treatment with a broad spectrum of antibacterial properties, and HIV infection. Difficulties associated with the treatment of patients infected with *C*. *albicans* compel the search for new therapeutic solutions.

The *S*. *hermaphrodita* extract obtained by us shows effective antifungal activity against the clinical strain *C*. *albicans* and moderate activity in relation to other *Candida* strains tested. The seed extract, which does not exert a cytotoxic or cytopathic effect on fibroblasts will meet the requirements for further testing stages as a potential anti-*Candida* preparation for the treatment of different types of candidiasis. Cytotoxicity to normal fibroblasts is an important feature in the evaluation of a new preparation with antifungal activity. The seed extract with anti-*C*. *albicans* activity characterized in this research, was granted on April 2, 2019 with a patent from the Patent Office in Poland (No 231698)^51^. Therefore, the extract is suitable for further biomedical research as a bioactive preparation and is a good candidate as an antifungal drug for the treatment of skin or systemic candidiasis.

## Materials and Methods

### Microorganisms

For the analysis of the action of the *S*. *hermaphrodita* extract on fungal cells three *Candida* strains were used: *C*. *albicans* a wild-type clinical isolate (from the collection of Department of Immunobiology of UMCS), *C*. *albicans* ATCC 10231, and *C*. *krusei* (*Issatchenkia orientalis)* ATCC 6258^40^. The yeast cells were cultured for 24 h in YPD medium (1% yeast extract, 2% peptone, 2% dextrose) at 28 °C with shaking at 200 rpm.

### Plant material

Seeds of *S*. *hermaphrodita* (L.) Rusby (*Malvaceae*), used in the experiments, came from commercial plant cultivation in Poland.

### Preparation of the *S. hermaphrodita* crude seed extract, dialysed seed extract and the fractions

To prepare the *S*. *hermaphrodita* extract the previously described method^40^ with minor modifications was used. After washing with water and 70% alcohol, the seeds of *S*. *hermaphrodita* were homogenised with Sörensen buffer (pH 6.0). The samples were then placed in liquid nitrogen for 10 min, and then in an ice bath for the same amount of time, and re-homogenised three times interchangeably. To obtain crude seed extract (CSE), the seed homogenate was centrifuged at 14,000 rpm for 10 min and filtered through a 0.22 μm pore size filter (Millipore).

To receive dialysed seed extract (DSE) containing compounds with molecular mass above 14 kDa, the CSE was dialyzed in water (in dialysis tubing with cut-off 14 kDa). The fractions were obtained by ultrafiltration at 4000 G and 4 °C for 30 min of the DSE (at the protein concentration of 1 mg mL^−1^), using Amicon Ultra-4 Centrifugal Filters (Merck) with MWCO 30, 50 and 100 kDa. Stock solutions of CSE, DSE and the fractions were prepared by dissolving freeze-dried samples in sterile deionized water. The samples dissolved completely forming a heterogeneous solution. The concentration of protein in the extracts was determined using the Bradford method^52^.

### Metabolic activity of Candida cells after treatment with the extracts

The metabolic activity of wild type *C*. *albicans*, *C*. *albicans* ATCC 10231, and *C*. *krusei* ATCC 6258 cells was determined by using LIVE/DEAD Yeast Viability Kit F-7030 FUN 1 (Life Technologies), described in our previous papers^31,40,53,54^. The *C*. *albicans* wild-type strain suspension was incubated with the CSE and DSE (at protein concentrations of 12.5, 25, 50, 100, and 200 μg mL^−1^), and the DSE fractions (each at protein concentrations of 25, 50 and 100 μg mL^−1^). The *C*. *albicans* ATCC 10231, and *C*. *krusei* ATCC 6258 cells were incubated with the DSE at protein concentrations of 12.5, 25, 50, 100, and 200 μg mL^−1^. After 72 h of incubation at 37 °C, each suspension of *Candida* cells were centrifuged for 5 minutes at 10,000 rpm at room temperature. Next, the cells were resuspended in 50 µL of 2% D-glucose solution containing 10 mM Na-HEPES (Sigma), pH 7.2 (GH). 30 µL of the *C*. *albicans* suspension in the GH solution were added to 30 µL of the GH-FUN solution. The samples were incubated for 30 minutes at 30 °C before microscopic observation. The experiment was done in triplicate.

The minimum inhibitory concentrations (MICs) for the *Candida* strains were determined with the broth microdilution method as specified by the Clinical and Laboratory Standards Institute (CLSI)^55^ and described by Fiołka and collaborators^56^. The CSE, DSE and DSE fractions with protein concentrations ranging from 6.25 to 100 µg mL^−1^ were analyzed. Cell growth was measured using a Benchmark Plus spectrophotometer at 600 nm (Bio-Rad). The experiment was repeated three times. The wild-type clinical isolate of *C*. *albicans* was used for further microscopic analysis.

For identification of apoptosis and necrosis, the *C*. *albicans* cells were stained with a mixture of fluorescent dyes Hoechst 33342 (Sigma) and propidium iodide (Sigma), respectively. The staining mixture was added to a yeast suspension in a 1:1 ratio and incubated for 5 min at 37 °C in the dark. Morphological analysis was performed under a fluorescence microscope Zeiss LSM 5 Pascal. Cells undergoing apoptosis demonstrated blue fluorescence of fragmented nuclei. Cells exhibiting pink fluorescent nuclei were interpreted as necrotic^40,54^.

### Preparation for microscopy techniques

The effect of the CSE on *C*. *albicans* was examined in cultures of *C*. *albicans* in liquid YPD poor medium according to Vilcinskas and Matha^57^ as reported previously^40^. 2.5; 5; 10, and 20 μL of the CSE (at final protein concentrations of 25, 50, 100, and 200 μg mL^−1^) was added to 100 µL of YPD poor medium containing 30 µL of *C*. *albicans* culture (1.9–2.0 × 10^9^ cells) and streptomycin sulphate (Sigma) at final concentration of 0.17 mg mL^−1^. The samples were filled up with YPD poor medium to a final volume of 200 µL and incubated for 3 days at 37 °C with gentle shaking. After that time, the antifungal action of the CSE was analyzed using different microscopy techniques. To analyse the effect of the DSE and the fractions against *C*. *albicans*, the same procedure was used.

### Fluorescence and differential interference contrast (DIC) microscopy

The morphology of *C*. *albicans* cells was analyzed in the cultures after staining and without staining using differential interference contrast (DIC) (Zeiss/Axiovert 200 M). After incubation with the CSE at protein concentrations of 25, 50, and 100 μg mL^−1^, the liquid cultures of *C*. *albicans* and the control culture were stained with Calcofluor White (Fluka) for 10 min in the dark^58^. The *C*. *albicans* cells were observed at 1000 x magnification (Zeiss/LEO LEO 912AB)^40,54^.

### Scanning Electron Microscopy (SEM)

*C*. *albicans* cells from a control culture and cells after incubation with the CSE at protein concentrations of 25, 50, and 100 μg mL^−1^ were fixed with 4% glutaraldehyde (Ubichem) in 0.1 M phosphate buffer, pH 7.0. Then the cells were dehydrated stepwise in a series of acetone (Sigma), dried using silica gel beads for 24 hours, and coated with gold using a K550X sputter coater (Quorum Technologies). The morphology of *C*. *albicans* cells was documented and analyzed using a Vega 3 scanning electron microscope (Tescan) with 10,000 x magnification^40^.

### Atomic force microscopy (AFM)

Analysis of the surface of the *C*. *albicans* cells after incubation with the CSE was carried out using AFM (Analytical Laboratory, Faculty of Chemistry, UMCS, Lublin, Poland) according to the protocol described by Fiołka *et al*.^59^. All measurements were carried out in contact and tapping operation modes using a NanoScope V AFM (Veeco Instruments Inc., Santa Barbara, CA, USA) equipped with NanoScope 8.10 software and a piezo-scanner with a maximum scan range of 150 μm × 150 μm.

### Determination of the cytotoxic activity

The cytotoxic effect of the CSE was measured using normal human skin fibroblast primary line (HSF). In the current protocol, each cell line was inoculated at a density of l0^4^ cells per mL on a microtiter plate in the RPMI medium. The tested component was then added at a final protein concentration of 100 μg mL^−1^and the cultures were incubated for 24, 48, and 72 hours under standard conditions (RPMI medium with 10% FBS, 5% CO_2_, 37 °C, 90% humidity)^56^. After the incubation, the medium was removed, and 25 µL MTT (5 mg mL^−1^ Sigma) were added per well. The culture was then incubated for 3 hours under the conditions described above. The absorbance of the solutions was measured spectrophotometrically (BIO RAD Model 680 XR) at 540 nm, using a reference wavelength of 690 nm and a plate reader. All experiments were done in triplicate. The images were documented using a Carl Zeiss Axiovert 40 CFL microscope^40^.

### Polyacrylamide gel electrophoresis

SDS–polyacrylamide gel electrophoresis (SDS–PAGE) was performed with the Laemmli method^60^ in 10% acrylamide gels. The DSE was analyzed for protein and sugar profile. A sample containing 15 µg of protein was used for protein detection and 3 µg of protein for detection of carbohydrate. The samples were heated at 100 °C for 3 min in the sample buffer. Protein bands were detected by staining with Coomassie Brilliant Blue R-250 (Sigma). Molecular weight markers (Bio-Rad) (SM0431) were used. Carbohydrate compounds were detected by staining with silver nitrate following sodium periodate oxidation^56,61^.

### Raman spectroscopy of the DSE

The protein secondary structure of the DSE was determined using Raman spectroscopy. The Raman spectroscopy analyses were conducted with an inVia Renishaw system. During measurements a laser emitting at a wavelength of 785 nm and a 1200 l/mm diffraction grating were used. At the beginning of each spectroscopic analysis, the spectroscope was calibrated using the Raman band of a silicon internal reference sample occurring at the 520.7 cm^−1^. Raman band of a silicon internal reference sample. Thirty Raman spectra of the study material were collected in the spectral range from 200 to 3200 cm^−1^. The spectroscopic measurements were carried out at room temperature (about 23 °C). The background in the Raman spectra was subtracted using the polynomial curve. The intensity of bands allowed the determination of the percentage of particular secondary structures of proteins. The intensity of bands corresponding to the alpha helix, beta sheet, beta turn and random coil structures was obtained by the curve-fitting process of the amide I band in the range between 1620 cm^−1^ and 1710 cm^−1^. Similar spectroscopic analysis of the protein secondary structure has been shown in our work^56^.

### ATR-FTIR analysis of the DSE

The Fourier Transform-Infrared Spectroscopy (FTIR), an analytical technique was employed to analyze the DSE. The DSE analyses were carried out using an FTIR Bruker ALPHA spectrometer at room temperature directly on the surface of the sample by the ATR technique at a mid-infrared spectral range. The DSE spectrum was compared with standard spectra in computer databases - ATR Pharmaceutical ST Japan.

### MALDI and LC-ESI-MS/MS analysis of the CSE and DSE fractions

The intact spectra of the CSE were registered with the use of MALDI TOF/TOF 5800 (ABSciex, Framingham, MA, USA). Sinapinic acid (SA, Sigma-Aldrich) and a binary matrix superDHB (DHB and 2-hydroxy-5-methoxybenzoic acid mixture, sDHB, Sigma-Aldrich) were used as a matrix. Lyophilized seed extract was used in LC-ESI-MS/MS analysis.

Overnight digestion in a trypsin solution was carried out according to the protocol described by Gundry *et al*.^62^ after reduction of the sample with dithiothreitol (Sigma-Aldrich, St Louis, USA) and alkylation with iodoacetamide (Sigma-Aldrich, St Louis, USA). The prepared sample was separated with the Ekspert MicroLC 200 Plus System (Eksigent, Redwood City, CA, USA) on the Eksigent microLC column ChromXP C18CL (3 µm, 120 Å, 150 × 0.3 mm) using a following 30 min gradient program: (1) 0−2 min − 10% solvent B, (2), 2–23 min – 10–90% solvent B, (3), 23−28 min − 90% solvent B, and (4) 28.1−30 min − 10% solvent B, where solvent A was 0.1% formic acid in water and solvent B 0.1% formic acid in acetonitrile. The eluate from the column was analyzed in a positive ion mode on a TripleTOF® 5600 + hybrid mass spectrometer equipped with DuoSpray Ion Source (SCIEX, Framingham, MA, USA).

The microLC-MS/MS system was controlled by the AB SCIEX Analyst TF 1.6 software. The experiments were performed in the data-dependent mode using settings described by Lewandowska *et al*.^63^. The acquired MS/MS spectra were searched against the Malvales Uniprot protein database in ProteinPilot® 4.5 Software (SCIEX) using the Paragon algorithm with an automated false discovery rate analysis.

### NMR analysis of the CSE

5 mg of sample was dissolved in 0.6 mL of 99.99% ^2^H_2_O. 1 H NMR spectrum was recorded at 30 °C with a Bruker Avance III 500 MHz spectrometer. Chemical shifts were referenced to acetone (δH 2.225).

### GC-MS analysis of the DSE

Sugar analysis was performed to identify and quantify monosaccharides in the sample of the DSE. The sample (~0.5 mg) was hydrolysed with 4 M trifluoroacetic acid (120 °C, 2 h), reduced with sodium borohydride and acetylated with acetanhydride in the presence of NaOAc. The obtained alditol acetates derivatives were analysed with system^64^.

### Statistical analysis

The results are expressed as mean SD (standard deviation). Normal distribution of data was examined using the one-way ANOVA with Dunnett’s multiple comparison test. A P-value less than 0.01 was considered statistically significant. Statistical analysis was performed using Statistica.

## Supplementary information

Supplementary information

Dataset 1

Dataset 2

## Data Availability

All data generated or analyzed during this study is included in this article (and its Supplementary Information files).
