# Supplementary material for: Sida hermaphrodita seeds as the source of anti - Candida albicans activity
Source: Sci Rep. 2019 Aug 22;9:12233. doi: 10.1038/s41598-019-48712-1 (PMC6706583; doi:10.1038/s41598-019-48712-1)
Supplement: Supplementary file 1 — Supplementary information [file 41598_2019_48712_MOESM1_ESM.pdf]

Sida hermaphrodita* seeds as the source of a*nti* - *Candida albicans activity

Kinga Lewtak, Marta J. Fiołka, Paulina Czaplewska, Katarzyna Macur, Zbigniew Kaczyński, Tomasz Buchwald, Ewa Szczuka, Jolanta Rzymowska

Supplementary information on electrophoresis results shown in Figure 9:

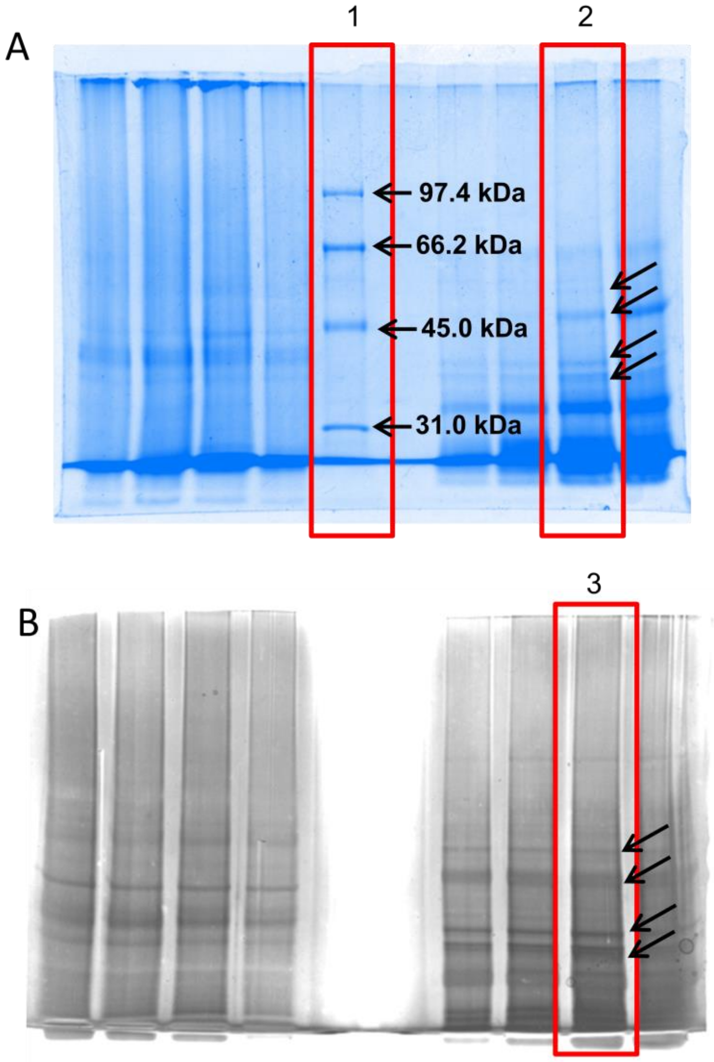

Gels showing the electrophoretic separation (SDS/PAGE) of the *Sida hermaphrodita* seed extract after dialysis stained with: A – Coomassie Brilliant Blue R-250 (Sigma) (protein staining); B - silver nitrate (carbohydrate staining). The red frames indicate the lines used in the Figure 9 of the submitted manuscript.: 1 –molecular weight markers (Bio-Rad); 2 – protein bands after analysis of sample containing 15 µg of protein; 3 – carbohydrate bands after analysis of sample containing 3 µg of protein. The arrows indicate the bands of carbohydrates and proteins located in the same place on the gel.

Legends of supplementary tables (datesets attached as excel files) :

Tab. 1. Suppl. List of all proteins identified in the crude *S. hermaphrodita* seed extract (CSE) provided with their identification parameters Unused ProtScores, numbers of peptides (95% confidence), total and percent of sequence coverage.

Tab. 2. Suppl. List of all proteins identified in the fractions of dialysed *S. hermaphrodita* seed extract provided with their identification parameters: Unused ProtScores, numbers of peptides (95% confidence), total and percent of sequence coverage.
